# Yeast Prions: Protein Aggregation Is Not Enough

**DOI:** 10.1371/journal.pbio.0020125

**Published:** 2004-04-13

**Authors:** Michael Y Sherman

## Abstract

Although many proteins -- both damaged and normal -- have a tendency to aggregate, only some are capable of dividing and propagating. What does it take to turn a protein aggregate into an infectious prion?

Many damaged and mutant polypeptides, as well as some normal proteins, have a tendency to aggregate in cells. Some protein aggregates are capable of “dividing” and propagating in cells, leading to formation of similar aggregates in daughter cells or even in neighboring cells due to “infection.” These self-propagating protein aggregates are called prions and constitute the basis of prion diseases. The infectious agent in these diseases is an abnormal conformation of the PrP protein (PrP^Sc^), which makes it protease-resistant and initiates its aggregation (Prusiner 1998). The abnormal aggregated species can recruit normal soluble PrP molecules into aggregates, thus inactivating them. The aggregates of PrP^Sc^ can proliferate within cells and be transmitted to other cells and tissues, leading to the spread of neurotoxicity.

## Prion Domains

**Figure 1:**
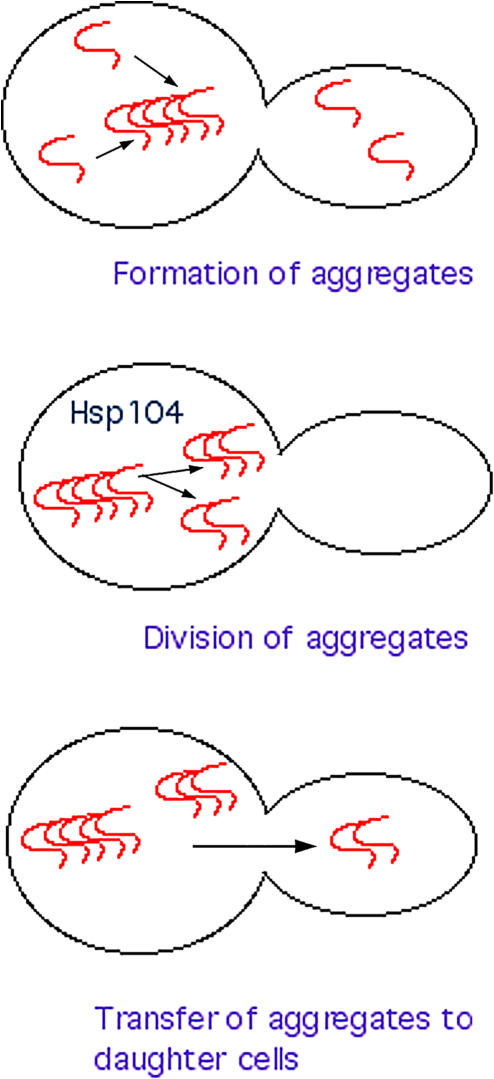
Aggregation, Division, and Transfer of Prions in Yeast

While so far only one prion protein is known in mammals, several prion-like proteins capable of forming self-propagating aggregates have been found in various yeast species. The common structural feature of yeast prion proteins is the so-called prion domain, characterized by the high content of glutamines (Q) and asparagines (N) (DePace et al. 1998; Michelitsch and Weissman 2000), also known as the Q/N-rich domain. The prion domains are the major structural determinants that are solely responsible for the polypeptide aggregation and propagation of the aggregates. Interestingly, the mammalian PrP^Sc^ is fundamentally different from yeast prions, since it lacks a Q/N-rich domain, indicating that distinct structural features are responsible for its ability to form self-propagating aggregates. The Q/N-rich domains in yeast prions are transferable in that, when fused to a heterologous polypeptide, they confer prion properties to this polypeptide. With a low probability, soluble proteins with prion domains can change conformation to form self-propagating aggregates, which can be transmitted to daughter cells (Lindquist 1997) (Figure 1). As with PrP^Sc^, yeast prions efficiently recruit soluble molecules of the same species, thus inactivating them (Lindquist 1997; Chernoff 2001; Wickner et al. 2001). Also with low probability, the aggregation-prone conformation of yeast prion proteins can reverse to a soluble functional conformation. Certain yeast prion proteins, when in soluble conformation, function in important pathways; e.g., Sup35 (forming [PSI^+^] prion) controls termination of translation, and Ure2 (forming [URE3^+^] prion) controls some membrane transporter systems. Aggregation of these proteins leads to phenotypes (e.g., suppression of nonsense mutations or transport defects) inherited in a non-Mendelian fashion owing to the nonchromosomal basis of the inheritance.

## Inheriting Variations

A remarkable feature of yeast prion proteins is their ability to produce distinct inherited “variants” of the prion. For example, [PSI^+^] prion could exist in several distinct forms that suppress termination of translation to different degrees. These “variants” of yeast prions are analogous to different prion “strains” of PrP^Sc^, which cause versions of the disease with different incubation periods and different patterns of brain pathology. The molecular nature of distinct PrP^Sc^ strains is determined by specific stable conformations of PrP. Similarly, “variants” of yeast prions are explained by different stable conformation states of the corresponding prion proteins (Chien et al. 2003). Strict conformation requirements for aggregate formation can also explain interspecies transmission barriers, where prion domains of Sup35 derived from other yeast species cannot cause formation of [PSI^+^] prion in Saccharomyces cerevisiae, in spite of a high degree of homology. This observation is very intriguing, especially in light of a recent finding that prion conformation of some proteins is required for formation of prions by the other proteins. For example, for de novo formation of [PSI^+^] prion, a distinct prion [RNQ^+^] should be present in a cell (Derkatch et al. 2001; Osherovich and Weissman 2001), probably in order to cross-seed Sup35 aggregates. This is in spite of relatively limited homology between the prion domains of these proteins. The apparent contradiction between the interspecies transmission barriers of very homologous prion proteins and possible cross-seeding of aggregates by prion proteins with more limited homology represents an interesting biological problem. On the other hand, this apparent contradiction may indicate that prion formation is a more complicated process than we currently think and that it may involve many cellular factors.

## What Do Prions Do?

Although yeast prions have been studied for almost ten years, very little is known about their biological significance. We do not know the functions of the majority of proteins that can exist as prions. Even if a function of prion proteins, such as with Sup35 or Ure2, is known, we do not understand the biological significance of their “prionization,” i.e., that they aggregate and propagate in the aggregated form. A very intriguing and unexpected finding was that formation of [PSI^+^] prion causes a wide variety of phenotypic alterations, which depend on the strain background (True and Lindquist 2000). In fact, comparison of yeast strains of different origin, each with and without [PSI^+^] prion, showed that certain strains with [PSI^+^] prion have different sensitivity to stresses and antibiotics than their non-prion derivatives, despite their genetic identity. In some strains, cells with [PSI^+^] prion demonstrated better survival than their non-prion counterparts in the presence of inhibitors of translation or microtubules, heavy metals, low pH, and other deleterious conditions, which of course gives a strong advantage to the [PSI^+^] cells. It is likely that some genomic mutations could be suppressed and therefore become silent when termination of translation by Sup35 is partially inactivated in [PSI^+^] prion cells (Lindquist 2000; True and Lindquist 2000). [PSI^+^] could also reveal previously silent mutations or their combinations. It was hypothesized that switches between prion and non-prion forms of Sup35 enhance survival in fluctuating environments and provide a novel instrument for evolution of new traits.

## Q/N Does Not Necessarily a Prion Make

Searching genomes of various species demonstrated that a relatively large fraction of proteins (between 0.1% and 2%) contain Q/N-rich domains (Michelitsch and Weissman 2000) or polyQ or polyN sequences. These domains are often found in transcription factors, protein kinases, and components of vesicular transport. The Q/N-rich domains usually are not evolutionary conserved and their functional role is largely unknown. Some of the Q/N-rich or polyQ domains facilitate aggregation of polypeptides, especially if expanded owing to mutations. Such expansion of the polyQ domains in certain neuronal proteins could cause neurodegenerative disorders, e.g., Huntington's disease or several forms of ataxia. Importantly, aggregates formed by polypeptides with the Q/N-rich or polyQ domains are not necessarily self-propagating aggregates, i.e., prions. In fact, there are additional structural properties of the polypeptides that provide the self-propagation (see below). Even if a protein with a polyQ domain does not form a prion, its aggregation may depend on certain prions. For example, recent experiments demonstrated that [RNQ^+^] prion dramatically stimulated aggregation of fragments of recombinant human huntingtin or ataxin-3 with an expanded polyQ domain cloned in yeast (Osherovich and Weissman 2001; Meriin et al. 2002). [RNQ^+^] facilitated the nucleation phase of the huntingtin fragment aggregation, suggesting that this prion can be directly involved in seeding of the aggregates. The major question now is whether there are analogous prion-like proteins in mammalian cells that are involved in aggregation of huntingtin or ataxin-3 and subsequent neurodegenerative disease.

The first indication that mammalian proteins with Q/N-rich domains can form self-propagating prions came from recent work with a regulator of translation cytoplasmic polyadenylation element-binding protein (CPEB) from *Aplysia* neurons (Si et al. 2003). The neuronal form of this protein has a Q/N-rich domain similar to the prion domains of yeast prions. The Q/N-rich domain from CPEB (CPEBQ), when fused to green fluorescent protein (GFP), conferred upon it prion-like properties. The CPEBQ–GFP fusion polypeptide existed in yeast cells in one of the three distinct states, i.e., soluble, many small aggregates, or few large aggregates. Mother cells almost always gave rise to daughter cells in which the CPEBQ–GFP polypeptide was in the same state, indicating the ability of these aggregates to be inherited, i.e., to self-propagate. Furthermore, full-length *Aplysia* CPEB protein, when cloned in yeast, can also exist in two distinct states, soluble and aggregated, which is an inherited feature. Very unexpectedly, unlike other prions, the aggregated state of CPEB was more functionally active than the soluble form (Si et al. 2003). These data strongly suggest that metazoan proteins with Q/N-rich domains are potentially capable of forming prions. The challenge now will be to establish whether CPEB can exist as a self-propagating aggregate in *Aplysia* or mammalian neurons.

## Mystery of Propagation

What makes protein aggregates in yeast propagate? The key cellular element that is critical for this process is molecular chaperone Hsp104 (Chernoff et al. 1995). This factor is specifically required for maintenance of all known prions within generations and probably is not involved in prion formation (i.e., initial protein aggregation). [PSI^+^] yeast cells have about 60 seeds of this prion (although this number differed in different [PSI^+^] isolates), and maintenance of about this number of seeds after cell divisions requires functional Hsp104 (Eaglestone et al. 2000). In fact, in the absence of Hsp104, prion aggregates continue to grow without increase in number and are rapidly lost in generations (Wegrzyn et al. 2001). Since this chaperone can directly bind to protein aggregates and promote there disassembly (Glover and Lindquist 1998), it was suggested that the main function of Hsp104 in prion inheritance is to disaggregate large prion aggregates to smaller elements, thus leading to formation of new seeds (Kushnirov and Ter-Avanesyan 1998). Interestingly, although Hsp104 is conserved among bacteria, fungi, and plants, animal cells do not have this chaperone or its close homologs. Therefore, if yeast-type prions with Q/N-rich domains exist in animal cells, there should be alternative factors that disaggregate large prion aggregates into smaller species in order to keep the number of seeds relatively constant and thus maintain the prions.

**Figure 2:**
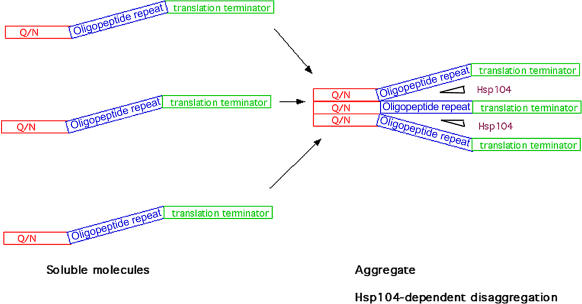
Distinct Domains of Sup35 Are Responsible for Aggregation and Division of Aggregates

The fact that some proteins with Q/N-rich domains form self-propagating aggregates, while others can aggregate but cannot form prions, suggests that there should be some structural elements either within the Q/N-rich sequence or close to it that confer the ability to propagate. In an article in this issue of *PLoS Biology* by Osherovich et al. (2004), the authors examined sequence requirements for prion formation and maintenance of two prion proteins, Sup35 and New1. They noted that both prion proteins contain an oligopeptide repeat QGGYQ in close proximity to Q/N-rich sequences and examined the functional significance of the repeats for aggregation and maintenance of the prions. In New1, in contrast to a deletion of the N-rich domain, deletion of the repeat did not affect aggregation of the protein or formation of the prion, but abrogated inheritance of the prion. With Sup35, the situation was somewhat more complicated, since repeats adjacent to Q/N-rich domain affected both protein aggregation and prion maintenance while more distant repeats affected only the prion inheritance. The authors suggested that the oligopeptide repeats facilitate the division of aggregates, either by serving as binding sites for Hsp104 or by altering the conformation of the polypeptides in aggregates to promote access for Hsp104 (Figure 2).

The likely possibility was that the oligopeptide repeats could be interchangeable between different prions, leading to creation of novel chimeric prions. In fact, the authors constructed an F chimera, a fusion protein having the N-rich domain of New1 and the oligopeptide repeat of Sup35. This fusion polypeptide efficiently formed prion [F^+^]. Furthermore, when the oligopeptide repeat sequence was added to a polyQ sequence, this fusion polypeptide also acquired the ability to form self-propagating aggregates. This work, therefore, clarifies the architecture of prions by defining two structural motifs in prion proteins that have distinct functions in aggregation and propagation. Interestingly, not all yeast prions have similar oligopeptide repeat motifs, indicating that distinct structures could confer prion properties to polypeptides that can aggregate. It would be important to identify these structures in order to understand the mechanisms of aggregate propagation. The work of Osherovich et al. (2004) may help to identify proteins from mammalian cells, plants, and bacteria that can potentially form prions. Finding these novel prions could be of very high significance since they may provide insight into a wide range of currently unexplained epigenetic phenomena.

## Abbreviations

CPEB: cytoplasmic polyadenylation element-binding protein
GFP: green fluorescent protein
